# A dual immune signature of CD8+ T cells and MMP9 improves the survival of patients with hepatocellular carcinoma

**DOI:** 10.1042/BSR20204219

**Published:** 2021-03-17

**Authors:** Huan Ding, Huan Hu, Feifei Tian, Huaping Liang

**Affiliations:** 1 School of Life Science and Engineering, Southwest Jiaotong University, Chengdu 610031, China; 2 State Key Laboratory of Trauma, Burns and Combined Injury, Department of Wound Infection and Drug, Daping Hospital, Army Medical University, Chongqing 400010, China

**Keywords:** CD8+ T Cells, Hepatocellular carcinoma, immune signature, MMP9, WGCNA analysis

## Abstract

The 5-year survival of hepatocellular carcinoma (HCC) is difficult due to the high recurrence rate and metastasis. Tumor infiltrating immune cells (TICs) and immune-related genes (IRGs) bring hope to improve survival and treatment of HCC patients. However, there are problems in predicting immune signatures and identifying novel therapeutic targets. In the study, the CIBERSORT algorithm was used to evaluate 22 immune cell infiltration patterns in gene expression omnibus (GEO) and the cancer genome atlas (TCGA) data. Eight immune cells were found to have significant infiltration differences between the tumor and normal groups. The CD8+ T cells immune signature was constructed by least absolute shrinkage and selection operator (LASSO) algorithm. The high infiltration level of CD8+ T cells could significantly improve survival of patients. The weighted gene co-expression network analysis (WGCNA) algorithm identified MMP9 was closely related to the overall survival of HCC patients. K-M survival and tROC analysis confirmed that MMP9 had an excellent prognostic prediction. Cox regression showed that a dual immune signature of CD8+ T cells and MMP9 was independent survival factor in HCC. Therefore, a dual prognostic immune signature could improve the survival of patient and may provide a new strategy for the immunotherapy of HCC.

## Introduction

Hepatocellular carcinoma is one of the common fatal malignant tumors in clinical [1]. It mainly developed from hepatitis and cirrhosis [2]. The early symptoms of HCC are relatively insidious, and most patients are already at an advanced stage when they are diagnosed [3]. Recently, new treatment methods have made some progress in the treatment of liver cancer [4]. However, the patient’s prognosis is still not satisfactory due to the high recurrence and metastasis of HCC [5]. Therefore, the determination of reliable prognostic markers is the key to improving the prognosis of HCC patients.

The tumor immune microenvironment is the environment where tumors interact with the immune system. The development of tumor was accompanied by mutual restriction between tumor and immune cells [6]. On the one hand, the immune system plays an antitumor effect by recognizing and killing tumor cells. On the other hand, tumor cells mainly promote tumorigenesis by immune suppression and immune tolerance [7]. With the rise of immunotherapy, immune checkpoint inhibitors (PD-1, CART, CTLA4, PD-L1 and PD-Ls) have made progress in the treatment and prognosis of HCC patients [8,9]. However, the disadvantage is that the treatment strategy is only applicable to a small number of patients. And there are problems in predicting immune signatures and identifying novel therapeutic targets. Studies revealed that the infiltration of TICs (tumor infiltrating immune cells) in malignant tumor cells is closely related to the expression of PD-1/PD-L1 [10,11]. The function and composition of TICs changed with the host immune status. Studies have reported the prognostic value of TICs in lung cancer, breast cancer and squamous cell carcinoma [12–14]. Accordingly, an extensive study of the immune infiltration pattern of TICs would help elucidate complex antitumor responses to improve the survival of HCC.

CIBERSORT [15] is a deconvolution algorithm based on a set of barcode gene expression, which is superior to other methods in the identification and of immune cells. The method can accurately quantify the level of infiltration of specific cell types. Moreover, the ESTIMATE can evaluate the tumor microenvironment's matrix abundance and tumor purity [16].

Our previous research has identified various biomarkers and potential inflammation-modulating therapeutic targets through experimental and bioinformatics method [17–19]. In the study, CIBERSORT was used to calculate the proportion of immune cells in GEO and TCGA database. A dual prognostic immune signature of TIC and IRG was constructed based on LASSO and WGCNA algorithms. Univariate and multivariate COX confirmed the potential value of the dual signature for improving the survival prediction of HCC.

## Materials and methods

### Collection and extraction of data

GSE76427 that contained 115 HCC samples and 52 normal samples came from GEO (https://www.ncbi.nlm.nih.gov/geo). The mRNA-seq (tumor = 371, normal = 50) expression data and corresponding clinical information were download in the TCGA database (https://cancergenome.nih.gov/; July 2020). The expression data was processed by log2 conversion. Samples with survival time < 1 day were excluded. The 2483 immune-related genes (Supplementary File S1) had been identified according to the ImmPort database (https://immport.niaid.nih.gov; July 2020). The overlapping IRGs were selected between the TCGA, GEO and ImmPort dataset.

### Analysis of immune fluctuation in HCC microenvironment

The CIBERSORT algorithm was used to calculate the proportion of 22 immune cells in each patient (perm = 1000). And the difference of immune cell infiltration were evaluated in tumor and normal tissues. The ESTIMATE algorithm was used to calculate the immune scores and matrix score of all samples. TIMER (https://cistrome.shinyapps.io/timer/) is used to analyze the correlation between prognostic IRG signature and immune cell infiltration.

### Construction of immune cell signature

The patient’s OS status and time was obtained from GEO and TCGA cohort. Univariate COX regression initially screened immune cells related to the patient’s life cycle (*P*<0.05). The LASSO model used a punishment mechanism to screen out prognostic signatures associated with the survival of the patient. The overlapping TICs signatures were selected between GEO and TCGA cohort. LASSO could be expressed as a constraint on the objective function.

$$\underset{\omega }{\min}\overset{m}{\underset{j=1}{\Sigma }}{\left(y_{j}-\overset{n}{\underset{i=1}{\Sigma }}\chi_{ji}\omega_{i}\right)}^{2},s.t.\overset{n}{\underset{i=1}{\Sigma }}|\omega_{i}|\leq \lambda$$

### WGCNA analysis

The expression matrix was established based on the overlapping IRGs of GEO data. The R package ‘WGCNA v1.69’ was used to build a co-expression network. The modules with potent immune characteristics were determined by calculating the correlation between each module and immune characteristics (*P*<0.05). The algorithm steps were as follows:

The Pearson algorithm was used to calculate the similarity between two genes, and then construct a correlation matrix.

$$\text{Y}=|{\text{S}}_{\text{ij}}|=\left|\frac{1+\text{cor}({\text{x}}_{\text{i}}+{\text{y}}_{\text{i}})}{2}\right|$$

A topological overlap matrix was created by calculating the adjacency matrix.

$$\text{A}={\text{a}}_{\text{ij}}=\text{power}({\text{S}}_{\text{ij}},\beta )=\;{\text{S}}_{\text{i}\text{j}}^{\beta }$$

$$\text{TOM}=\frac{\Sigma_{\text{k}\neq \text{ij}}{\text{A}}_{\text{ik}\cdot {\text{A}}_{\text{kj}}+{\text{A}}_{\text{ij}}}}{min(\Sigma_{k}{\text{A}}_{\text{ik}}+\Sigma_{\text{k}}{\text{A}}_{\text{jk}})+1-{\text{A}}_{\text{ij}}}$$

The module membership was built.

$$\text{ME}=\text{princomp}\left({\text{X}}_{\text{ij}}^{\text{q}}\right)$$

$${\text{MM}}_{\text{i}}^{\text{q}}=\text{cor}\left({\text{X}}_{\text{i}},{\text{ME}}^{\text{q}}\right)$$

### Evaluation of prognostic value

The prognostic performance of immune signature was evaluated by Kaplan–Meier survival and tROC curve. The ‘Correlation Analysis’ module of GEPIA2 was used to evaluate the correlation between IRG signature and immune cell marker genes. Univariate and multivariate Cox regression were used to analyze the immune characteristics and clinical parameters.

### Statistical

Bioinformatics analysis was executed by using R v3.6.1 and its corresponding packages. *P*<0.05 was considered statistically significant.

## Results

### Immune cell infiltration pattern of HCC

**Figure 1:**
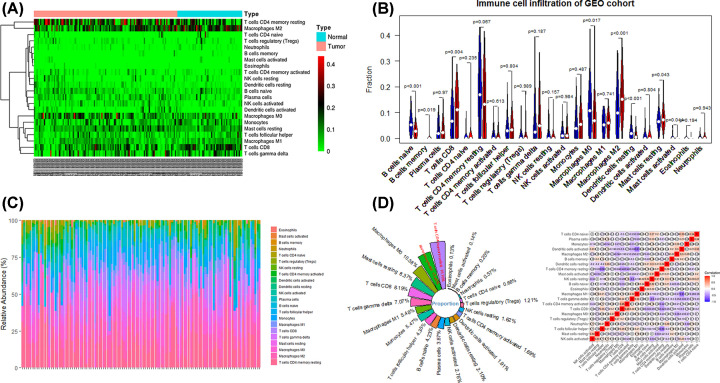
Analysis of 22 immune cell infiltration according to CIBERSORT algorithm (**A**) Heatmap of 22 immune cells. (**B**) Differences of 22 immune cells infiltration between the HCC and Normal group. (**C**) The percentage of immune cells in HCC sample. (**D**) Average infiltration rate and correlation analysis.

The CIBERSORT algorithm described the infiltration pattern of 22 immune cells in the GEO cohort and TCGA cohort. The immune score of each sample was calculated according to the ESTIMATE algorithm (Supplementary File S2). Heat map of the distribution of 22 immune cells in the GEO cohort (Figure 1A). B cells naive (*P*<0.001), B cells memory (*P*=0.019), T cells CD8 (*P*=0.004), Macrophages M0 (*P*=0.017), Macrophages M2 (*P*<0.001), Dendritic cells resting (*P*<0.001), Mast cells resting (*P*=0.043), Mast cells activated (*P*=0.044), there were significant infiltration differences between HCC and normal tissues (Figure 1B). The percentage of immune cells in each HCC sample was shown in Figure 1C. And the average infiltration rate of immune cells and the correlation matrix were shown in Figure 1D.

### Determination and prognostic significance of TIC signature

**Figure 2:**
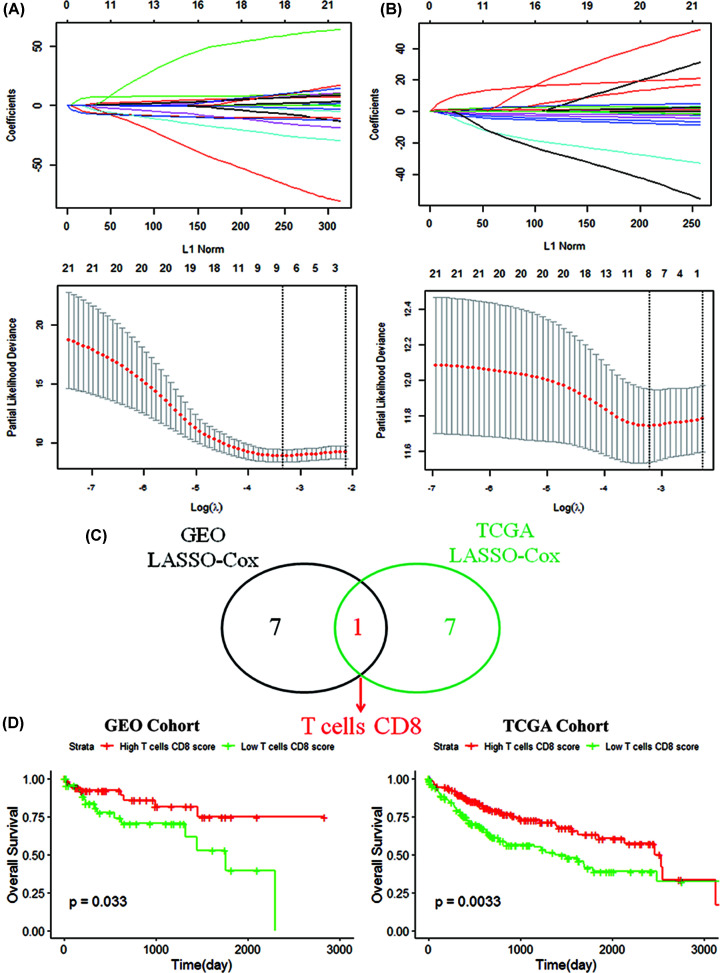
Definition and prognostic analysis of CD8+ T Cells (**A**) LASSO analysis identified eight TICs in GEO cohort. (**B**) Determine the minimum value of λ to be eight based on TCGA cohort. (**C**) Identification of CD8+ T cells. (**D**) Survival analysis of CD8+ T cells.

The eight TICs immune signatures were obtained based on univariate COX regression and LASSO analysis (Figure 2A,B). CD8+ T cells was identified by Venn analysis (Figure 2C). The survival curve showed that the high infiltration level of CD8+ T cells could significantly improve survival of HCC (Figure 2D).

### Identification of IRG modules related to TIC signature

**Figure 3:**
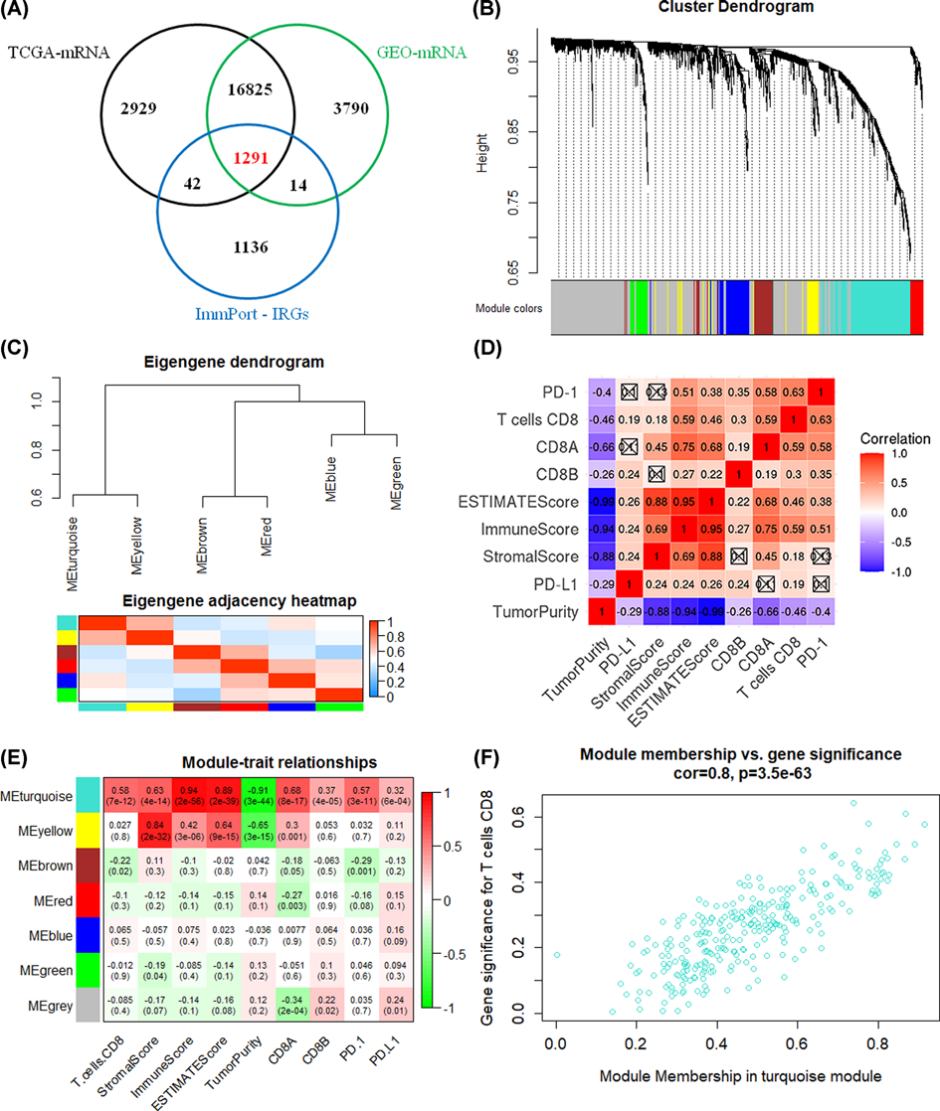
Identification of key IRGs based on WGCNA (**A**) 1291 overlapping IRGs from three databases. (**B**) A dendrogram of IRGs including gray modules (genes not classified into modules). (**C**) Module–module correlation analysis diagram. (**D**) Correlation analysis between CD8+ T cells and eight immune characteristics. (**E**) Correlation heatmap of modules and immune traits. The turquoise module showed outstanding correlation. (**F**) In-depth analysis of the correlation between turquoise module and T cell CD8.

The expression data of 1291 overlapping IRGs were screened to perform WGCNA analysis (Figure 3A). The six modules were identified by constructing a scale-free network (Figure 3B). The module correlation analysis demonstrated that turquoise strongly correlated with the yellow module (Figure 3C). CD8+ T cells was significantly correlated with eight immunophenotypic parameters (PD-1, PD-L1, CD8A, CD8B, ImmuneScore, ImmuneScore, ESTIMATEScore, TumorPurity) (Figure 3D). The turquoise module had a strong correlation with nine immunophenotypic parameters (Figure 3E). Further analysis indicated that the turquoise module were significantly correlated with CD8+ T cells (cor = 0.80, *P* = 3.5e-63) (Figure 3F). Therefore, the turquoise module (278 IRGs) was identified as a key module.

### Construction and prognostic value of immune IRG signature

**Figure 4:**
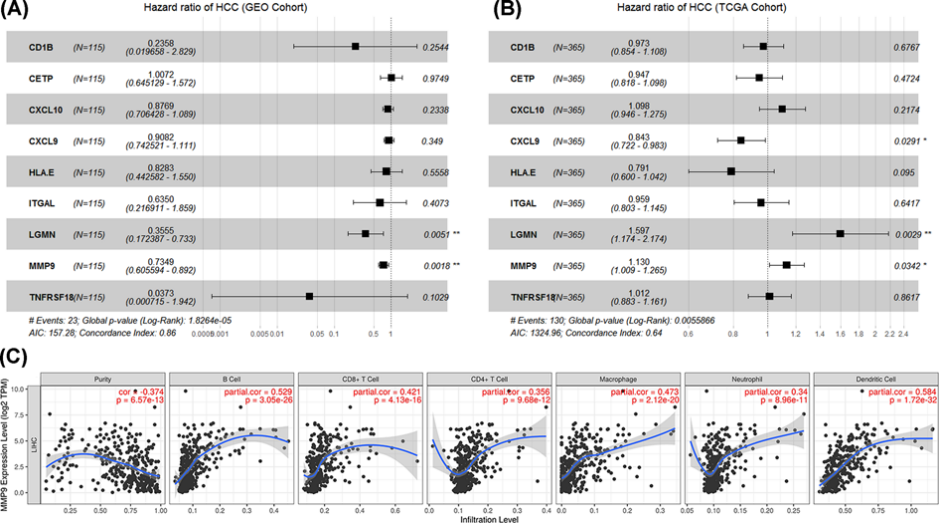
Construction of MMP9 signature (**A** and **B**) Multivariate COX regression of nine IRGs in GEO cohort and TCGA cohort. (**C**) Correlation analysis between the expression of MMP9 and immune cells based on TIMER.

The 36 IRGs were identified to be significantly related to the patient’s OS in the GEO cohort (*P*<0.05) through univariate COX regression analysis. Nine IRGs signatures were obtained by LASSO COX analysis (Supplementary Figure S1). MMP9 was identified as a prognostic immune signature based on GEPIA2 correlation analysis (Table 1 and Supplementary Table S1) and multivariate COX regression analysis (Figure 4A,B). TIMER evidenced that the high expression of MMP9 significantly increased the infiltration level of the B cells (cor = 0.529, *P* = 3.05e-26), CD8+ T cells (cor = 0.421, *P* = 4.13e-16), CD4+ T cells (cor = 0.356, *P* = 9.68e-12), macrophage (cor = 0.473, *P* = 2.12e-20), neutrophil (cor = 0.34, *P* = 8.96e-11), dendritic cells (cor = 0.584, *P* = 1.72e -32). Interestingly, the increased expression of MMP9 could reduce tumor purity (Figure 4C).

**Table 1:** Correlation analysis between gene signature and CD8A, CD8B

Gene	CD8A		CD8B	
	Cor	*P*.value	Cor	*P*.value
CD1B	0.24	3.2e−08	0.23	5.4e−08
CETP	0.16	2e−04	0.14	0.0011
CXCL10	0.45	0	0.41	0
CXCL9	0.72	0	0.64	0
HLA-E	0.48	0	0.45	0
ITGAL	0.49	0	0.43	0
LGMN	0.54	0	0.53	0
MMP9	0.18	4.6e−05	0.17	9.4e−05
TNFRSF18	0.22	2.8e−07	0.23	1.1e−07

**Figure 5:**
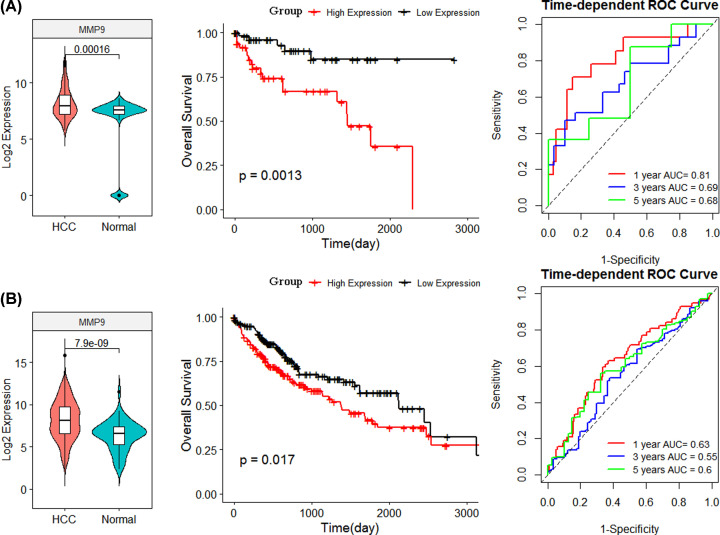
Prognostic value of MMP9 (**A** and** B**) Analysis of expression patterns, survival and tROC based on GEO cohort and TCGA cohort.

The GEO cohort found that the expression of MMP9 was significantly up-regulated in tumor tissues (*P*=0.00016). The high expression of MMP9 corresponded to the poor survival of HCC (*P*=0.0013). The tROC curve analysis evidenced that the MMP9 signature had an excellent prognostic predictive effect (Figure 5A). The TCGA cohort analysis results also confirmed that MMP9 had significant prognostic value (Figure 5B).

**Figure 6:**
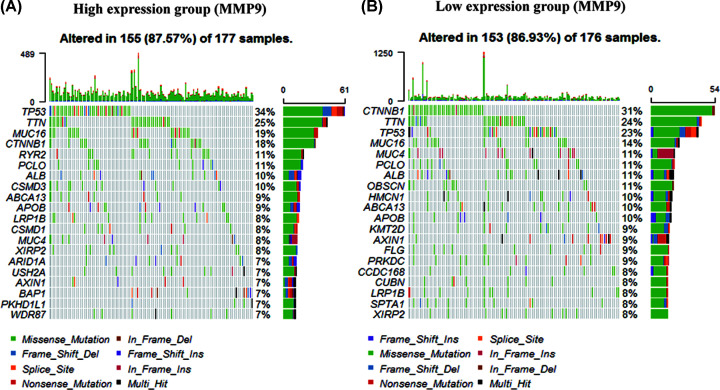
Correlation analysis between the MMP9 and HCC mutations from TCGA cohort (**A** and** B**) The mutation status of the top 20 genes in the high and low expression group.

We characterized the mutants of the TCGA cohort based on the expression of MMP9. The 20 genes were observed with the highest mutation frequency in the TCGA cohort. We found that basically all genes had higher mutation frequency in the high expression group than in the low expression group (Figure 6). All samples were dominated by mutations of TTN, TP53 and MUC16.

### The immune infiltration landscape of MMP9 in HCC

**Figure 7:**
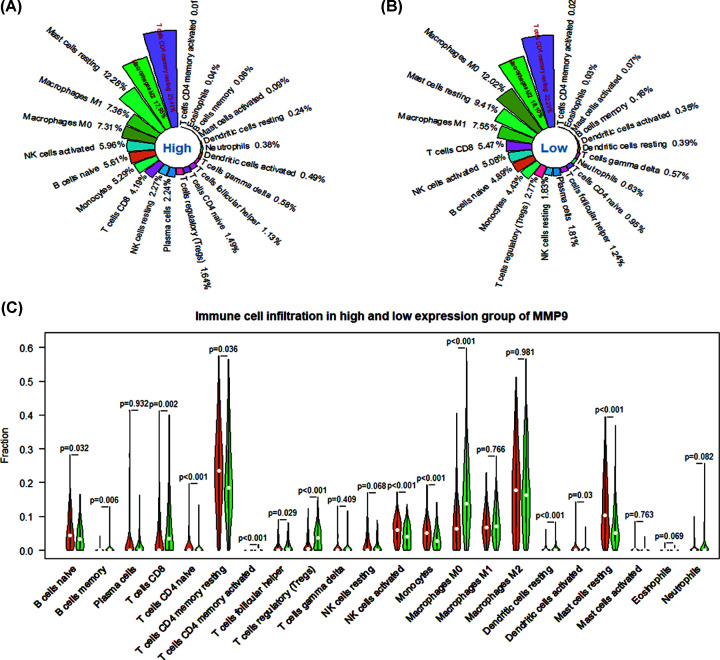
Immune infiltration levels in high and low expression groups of MMP9 (**A** and** B**) The infiltration proportion of 22 immune cells in the high and low expression groups. (**C**) Differences in the level of immune cell infiltration between the high and low expression groups.

To further confirm the immunity of MMP9, we evaluated the immune infiltration landscape of different expression groups based on TCGA data. We found that T cells CD4 memory resting (high = 23.41%, low = 22.23%), Macrophages M2 (high = 17.95%, low = 18.10%) and Macrophages M0 (high = 12.28%, low = 12.02%) were among the infiltration ratios more than 50% (Figure 7A,B). Compared with the low expression group, B cells naïve (*P*=0.032), B cells memory (*P*=0.006), T cells CD8 (*P*=0.002), T cells CD4 naïve (*P*<0.001), T cells CD4 memory resting (*P*=0.036), T cells CD4 memory activated (*P*<0.001), T cells follicular helper (*P*=0.029), T cells regulatory (Tregs) (*P*<0.001), NK cells activated (*P*<0.001), monocytes (*P*<0.001), macrophages M0 (*P*<0.001), dendritic cells resting (*P*<0.001), dendritic cells activated (*P*=0.03) and mast cells resting (*P*<0.001) had significant differences (Figure 7C).

### Independent prognostic analysis of TIC signature and IRG signature

**Figure 8:**
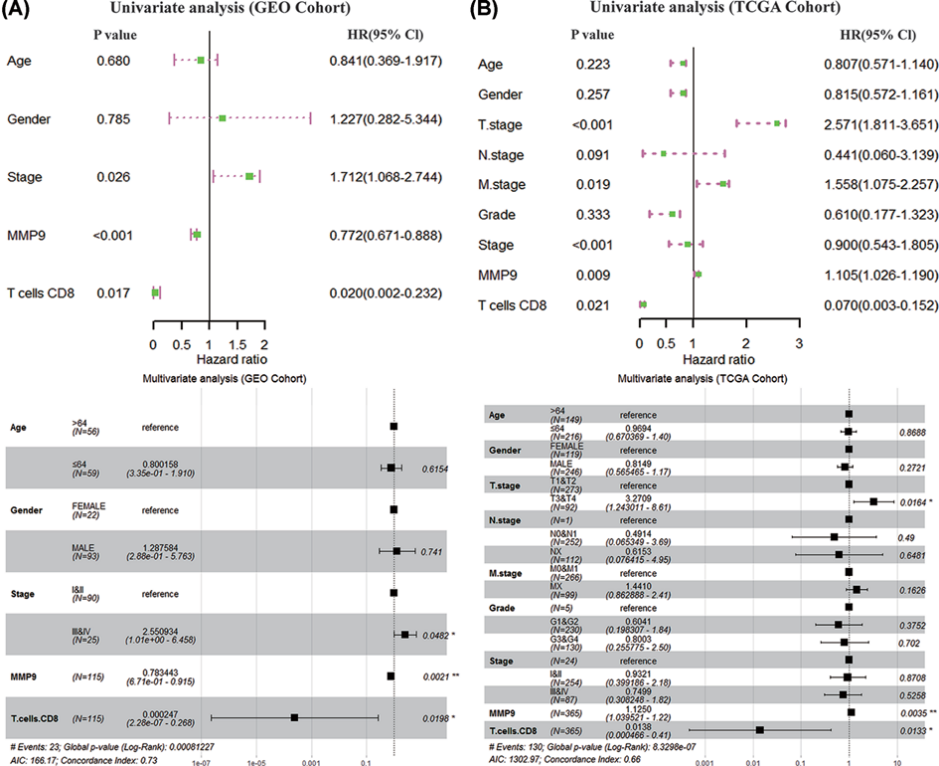
Independent prognostic analysis (**A** and** B**) Univariate and multivariate COX analysis based on GEO cohort and TCGA cohort.

The univariate COX regression model of GEO cohort indicated that a dual signature of CD8+ T cells (HR = 0.020, *P*=0.017) and MMP9 (HR = 0.772, *P*<0.001) was significantly related to the survival of HCC patients. Multivariate COX revealed that a dual signature of CD8+ T cells (HR = 0.000247, *P*=0.0198) and MMP9 (HR = 0.783443, *P*=0.0021) was an independent predictor of the prognosis of HCC (Figure 8A). Similarly, TCGA cohort analysis also confirmed this result (Figure 8B).

## Discussion

Tumor microenvironment played a critical role in the tumor progression. Abundant evidence shows that TICs play an essential role in predicting tumor progression and prognosis [20]. The distribution of TICs is significantly different between different tumor stages or different types of tumors. The evaluation of the number, phenotype and spatial distribution of TICs may provide reliable treatment strategies of patients. At present, most studies have focused on RNA signatures to predict prognosis and the role of TICs in the progression and treatment of HCC [21–25]. However, the predictive capability of single biomarker is usually defective. Conversely, the combination of immunotherapy and targeted therapy is expected to improve the survival after standard treatment in cancer. But the value of the dual signature combining TIC and IRG in improving the survival of HCC has not been studied yet. Therefore, in the preent study, a dual immune prognostic model was constructed through GEO, TCGA and ImmPort dataset, which may provide a new strategy for improving the survival and immunotherapy of HCC.

This study described the distribution of TICs in HCC based on CIBERSORT. LASSO analysis confirmed that only CD8+ T cells was significantly correlated with the overall survival of HCC. We found that the infiltration pattern of CD8+ T cells was significantly different between HCC and normal groups. High infiltration levels of CD8+ T cells could improve the prognosis of HCC. Besides, we studied the complicated relationship between T-cell CD8 and routine clinical parameters to reveal whether T cell CD8 is an independent variable of HCC. Interestingly, Cox regression analysis confirmed that CD8+ T cell is an independent prognostic factor of HCC. Sometimes CD8+ T cells killed cancer cells, but they promoted cancer cell proliferation occasionally. Most CD8+ T cells are activated and transformed into cytotoxic T lymphocytes to play a direct killing effect by recognizing TAA [26]. However, CD8+ T cells promotes the proliferation of HCC by maintaining immune tolerance [27]. Huang et al. reported that the expression of PD-L1 is significantly related to CD8+ T cells in HCC [28]. Guo et al. found that the expression of CD8 + T cells in HCC was significantly higher than in normal tissues [29]. CD8+ T cells is related to the progression and prognosis of HBV-HCC [30]. These studies indicate that T cell CD8 plays an essential role in the process of HCC. And it is a specific marker for evaluating the prognosis of HCC.

Cytotoxic T lymphocytes (CTL) in the HCC microenvironment are mainly CD8+ T cells [31]. Interferon (IFN)-γ produced by CD8+ T cells is a key factor in antitumor immunity. It can increase antigen presentation, proinflammatory cytokine production and directly kill tumor cells [32]. Preclinical studies have found that exposure of tumors to IFN-γ secreted by antigen-specific CD8+ T cells can lead to tumor cells' genetic instability. DNA damage response-related copy number variation, DNA editing, and DNA repair-related gene changes [33]. CD8+CXCR5+T in HCC produces IL-21 (interleukin-21), which induces B cells to differentiate into plasma cells that produce Ig G. And it plays a key role in the humoral immunity of HCC, which is related to a good prognosis [34]. HIF-α1 induced the increase of TREM-1 expression in TAMs, impairing the cytotoxic function of CD8+T cells and inducing apoptosis. And it blocks spontaneous and PD-L1 antibody-mediated anti-HCC effects [35]. The depleted CD8+ T cells seem to have evolved in the liver, and immunotherapy may restore the depleted T cells in the HCC [36]. Therefore, CD8 + T cells play an important role in the progression of HCC.

MMP9 is a member of the zinc-dependent endoprotease family. It is involved in processes such as inflammation, apoptosis and immunity [37]. Wang et al. found that high expression of MMP9 was significantly associated with HCC metastasis [38]. And The MMP9 expression was significantly correlated with the penetration level of TICs. Liu et al. confirmed that macrophages M2 affects the progression of HCC through MMP9 [39]. MMP9 acted actively to regulate the immune response and pathogenesis of HCC [40,41]. In our study, the survival curve, tROC curve and COX regression analysis confirmed that MMP9 was an independent indicator predicting the survival of HCC.

In conclusion, our study confirmed that a dual immune signature of CD8+ T cells and MMP9 could improve the survival of HCC. But the application potential of a dual immune signature in HCC immunotherapy still needs sufficient experimental verification.

## Supplementary Material

Supplementary File S1-S2

Supplementary Figure S1 and Table S1

## Data Availability

The datasets analyzed in this study are available in Gene Expression Omnibus (GEO) and The Cancer Genome Atlas (TCGA).

## Abbreviations

GEO: gene expression omnibus
HCC: hepatocellular carcinoma
IRG: immune-related gene
K-M: Kaplan–Meier
LASSO: least absolute shrinkage and selection operator
OS: overall survival
TCGA: the cancer genome atlas
TIC: tumor infiltrating immune cell
WGCNA: weighted gene coexpression network analysis
