# Supplementary material for: A dual immune signature of CD8+ T cells and MMP9 improves the survival of patients with hepatocellular carcinoma
Source: Biosci Rep. 2021 Mar 17;41(3):BSR20204219. doi: 10.1042/BSR20204219 (PMC7969702; doi:10.1042/BSR20204219)
Supplement: Supplementary Figure S1 and Table S1 [file BSR-2020-4219_supp1.pdf]

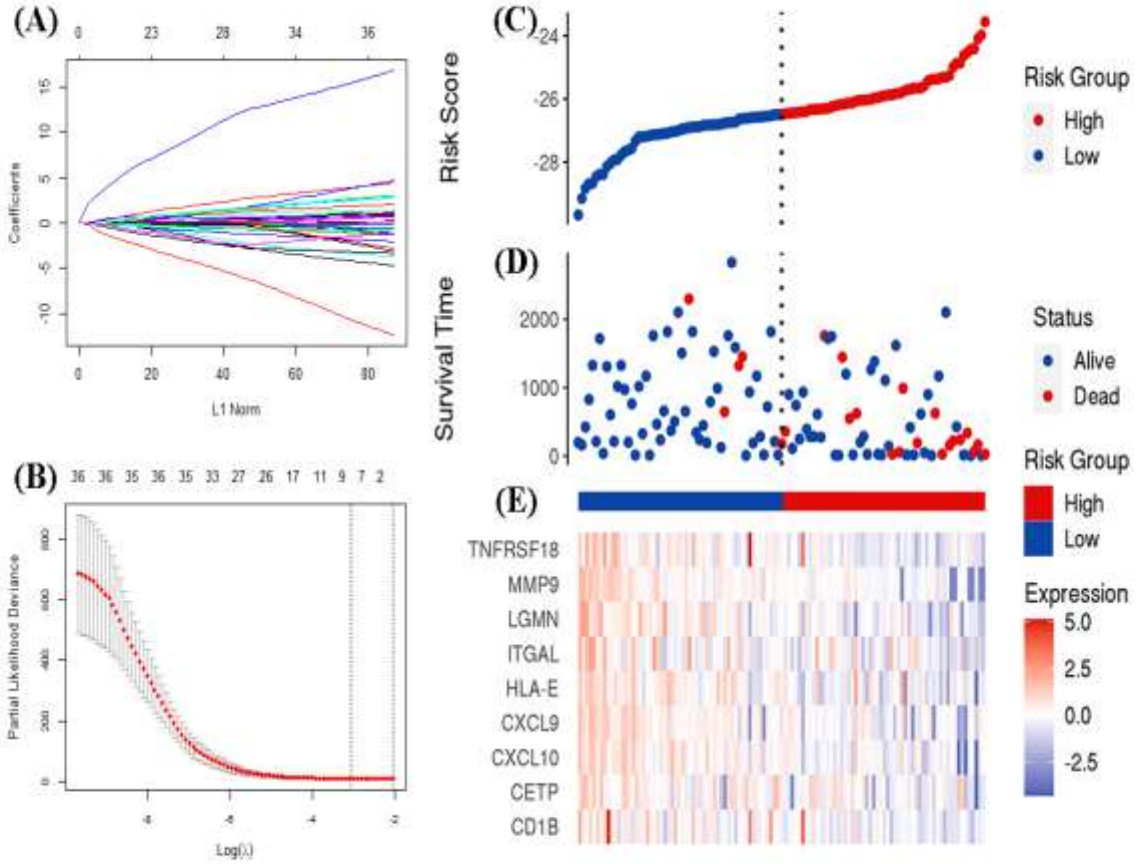
(A)
Coefficients
L1 Norm
(B)
Partial Likelihood Deviance
Log(λ)
(C)
Risk Score
Risk Group
High
Low
(D)
Survival Time
Status
Alive
Dead
(E)
TNFRSF18
MMP9
LGMN
ITGAL
HLA-E
CXCL9
CXCL10
CETP
CD1B
Risk Group
High
Low
Expression
5.0
2.5
0.0
-2.5

Table S1 Symbol and gene name correspondence list

Symbol	Gene name
CD1B	CD1b molecule
CETP	cholesteryl ester transfer protein, plasma
CXCL10	chemokine (C-X-C motif) ligand 10
CXCL9	chemokine (C-X-C motif) ligand 9
HLA-E	human leucocyte antigen, E
ITGAL	integrin alpha L
LGMN	legumain
MMP9	matrix metallopeptidase 9
TNFRSF18	TNF-receptor-superfamily-member 18
CD8A	CD8a molecule
CD8B	CD8b molecule
PD-1	programmed death 1
PD-L1	programmed death ligand 1
CART	chimeric antigen receptor T cells
CTLA4	cytotoxie Tlymphocyte associated antigen 4
